# Depression, antidepressants and fall risk: therapeutic dilemmas—a clinical review

**DOI:** 10.1007/s41999-021-00475-7

**Published:** 2021-03-15

**Authors:** E. P. van Poelgeest, A. C. Pronk, D. Rhebergen, N. van der Velde

**Affiliations:** 1 Department of Internal Medicine, Geriatrics, Amsterdam Public Health Research Institute, Amsterdam University Medical Center, Amsterdam, The Netherlands; 2 Amsterdam University Medical Center, Department of Psychiatry, Amsterdam Public Health Research Institute, Amsterdam University Medical Center, Amsterdam, The Netherlands; 3 Mental Health Care Institute GGZ Centraal, Amersfoort, The Netherlands

**Keywords:** Antidepressants, FRIDs, Falls, Deprescribing, Geriatric, Orthostatic hypotension

## Abstract

**Purpose:**

The aim of this clinical review was to summarize the existing knowledge on fall risk associated with antidepressant use in older adults, describe underlying mechanisms, and assist clinicians in decision-making with regard to (de-) prescribing antidepressants in older persons.

**Methodology:**

We comprehensively examined the literature based on a literature search in Pubmed and Google Scholar, and identified additional relevant articles from reference lists, with an emphasis on the most commonly prescribed drugs in depression in geriatric patients. We discuss use of antidepressants, potential fall-related side effects, and deprescribing of antidepressants in older persons.

**Results:**

Untreated depression and antidepressant use both contribute to fall risk. Antidepressants are equally effective, but differ in fall-related side effect profile. They contribute to (or cause) falling through orthostatic hypotension, sedation/impaired attention, hyponatremia, movement disorder and cardiac toxicity. Falling is an important driver of morbidity and mortality and, therefore, requires prevention. If clinical condition allows, withdrawal of antidepressants is recommended in fall-prone elderly persons. An important barrier is reluctance of prescribers to deprescribe antidepressants resulting from fear of withdrawal symptoms or disease relapse/recurrence, and the level of complexity of deprescribing antidepressants in older persons with multiple comorbidities and medications. Practical resources and algorithms are available that guide and assist clinicians in deprescribing antidepressants.

**Conclusions:**

(De-) prescribing antidepressants in fall-prone older adults is often challenging, but detailed insight in fall-related side effect profile of the different antidepressants and a recently developed expert-based decision aid STOPPFalls assists prescribers in clinical decision-making.

## Key summary points

**Aim:**

To summarize the existing knowledge on fall risk associated with antidepressant use in older adults, including the underlying pathophysiology, and assist clinicians in (de-) prescribing antidepressants.

**Findings:**

Untreated depression and antidepressant use both increase fall risk in older people. Antidepressants differ in fall-related side effect profile. They contribute to (or cause) falling through orthostatic hypotension, sedation/impaired attention, hyponatremia, movement disorders and cardiac toxicity. Although withdrawal of antidepressants is recommended in fall-prone elderly persons, physicians are frequently reluctant to deprescribe antidepressants. Practical resources and algorithms are available that guide and assist clinicians in deprescribing antidepressants.

**Message:**

Insight in fall-related side effect profile of antidepressants, and clinical decision tools such as the STOPPFalls antidepressant withdrawal algorithm assist prescribers in rational (de-) prescribing decision making.

## Introduction

In Western societies, over one-third of community dwelling and over 40% of institutionalized older persons fall every year [1]. The consequences of fall incidents in older people are potentially serious and besides the related injuries include decreased quality of life, loss of autonomy, risk of institutionalization and death. Twenty percent of older adults require medical attention for a fall, with 5–10% having a serious injury such as a head injury or a fracture [2]. Every year, ~ 40,000 fatal falls are reported in the European Union [3].

In general, falls are multifactorial. Over 400 fall risk factors have been identified, of which the most important modifiable risk factors are mobility problems and the use of fall-risk increasing drugs (FRIDs) [2]. With the total number of chronic diseases and the accompanying number medications rising with age, also the use of FRIDs increases [4]*.* Psychotropic medication, including antidepressants, has consistently been associated with falls. A recent systematic review and meta-analysis published by our group showed an odds ratio (OR) of 1.57 for antidepressant-related falls [5].

Depression is a common and important cause of morbidity and mortality in older people worldwide, affecting around 10–15% of community-dwelling older persons [6]. In the geriatric population, depression is associated with functional decline, cognitive impairment, premature death by suicide, increased (cardiovascular and all-cause) mortality, decreased quality of life and premature nursing home admission [7]. If left untreated, symptoms may persist for years [8]. Untreated depression is independently associated with increased fall risk: a meta-analysis showed an OR of 1.63 (95% CI 1.36–1.94) for depression-associated falling [1]. The pathophysiologic mechanisms underlying the association between depression and falling are complex. Major mechanisms are psychomotor retardation, deconditioning, gait and balance abnormalities, impaired sleep, and impaired attention. Often, multiple pathways interact and co-occur [9]. Also, excessive fear of falling contributes to increased fall risk in depressed older persons. It negatively influences gait and balance, and thereby increases tendency to fall [10].

Antidepressant use is also common in older persons; almost 10% of community-dwelling older people [6], and one-third of nursing home residents use antidepressants [11]. With age, adverse outcomes of antidepressant use, including premature death, are highly prevalent [6]. Due to reduced physiological reserve, and changes in pharmacokinetics and pharmacodynamics with aging or other conditions, older persons are more susceptible for medication-related side effects than younger adults [12].

Although often overlooked, fall incidents are among the most important adverse drug effects of antidepressants in the older population. Given the above-mentioned potential consequences of falls in older persons on morbidity and mortality, fall prevention is essential in this population. Medication review is a crucial element of the multifactorial fall assessment, especially if the patient uses psychotropic medicines. If clinical condition allows, withdrawal of psychotropic drugs is recommended [2]. The process of health care supervised medication withdrawal (or dose reduction) is called deprescribing [13]. It aims to improve patient outcomes by correcting or preventing medication-related complications, and reduce health care costs. The effect of deprescribing medications on fall risk was studied in several systematic reviews and meta-analyses [14, 15], showing that withdrawal of psychotropic medication reduced the number of falls of individuals (relative attributable risk (RaR) 0.34, 95%CI 0.16–0.73), but not the number of fallers.

As stated above, prevalence of both depression, accompanying antidepressant use and falls are common in older people. In an effort to prevent future falls and protect function and quality of life, judicious prescription of antidepressants is essential. In clinical practice, however, these (de-) prescribing decisions are often highly complex, highlighting the need for detailed knowledge of the risks and benefits of prescribing and deprescribing of this drug class. In this clinical review, we give an overview of the current literature regarding antidepressant use and antidepressant withdrawal in the light of fall risk. This clinical review was informed by a literature search conducted in May 2020 in Pubmed and Google Scholar with citation and reference checking. Personal reference libraries and antidepressant SmPCs (summary of product characteristics) were also utilized. Keywords for the searches included “falls”, “antidepressant”, “deprescribing” and “older adults” (and appropriate variations). We do not provide an exhaustive systematic review of the literature on all antidepressants and their relationship to falling, but merely focus on the most commonly prescribed drugs in geriatric depression. As such, antidepressants in general are discussed while examples of medication classes are provided to illustrate the potential fall-related safety concerns. We intend to call attention to the increased fall risk associated with both depression and antidepressant use in the aged. Also, we intend to assist clinicians in appropriate use of antidepressants in older persons with increased risk of falling.

## Medication review and reconciliation

### Match antidepressant use to an appropriate indication

The first step in performing a medication review aimed at reducing fall risk is to thoroughly review all current medications for their (contra-) indications, including the antidepressant history (efficacy, doses, intolerance, depression severity), use of over-the-counter drugs and as required medications, supplements, alcohol use and medications not taken orally or in solid form [16]. Fall risk increases with the number of FRIDs, and with combining FRIDs with alcohol [17]. Thus, combined use of antidepressants and benzodiazepines, other sedating drugs, antihypertensives and/or alcohol should be avoided because of the increasing risk of falling due to sedation, psychomotor impairment, drowsiness, and hypotension [17]. If sedation is required to treat -for instance- severe agitation and/or anxiety associated with suicidal tendency, combinations of centrally acting sedatives should be prescribed only for limited duration and in the lowest possible dose [18]. It is essential to evaluate not only why the antidepressant has been prescribed, but also whether antidepressant therapy is still indicated [19]. With aging, benefit/risk ratios often change over time, highlighting the need to regularly reassess the appropriateness of antidepressant therapy in older persons. Falls are among the circumstances that should trigger prescribers to perform a medication review in any older patient, and re-evaluate whether antidepressant use is still appropriate [2, 13].

Pharmacological treatment of depression is indicated in case of a major depressive disorder. In mild to moderate severity, psychotherapy is recommended as an initial treatment option [8]. Although psychotherapy is equally effective as treatment with antidepressants, and obviously devoid of adverse drug effects, it is underutilized in clinical practice due to among others the assumption that psychological help is not helpful and due to practical issues (e.g. costs) [20]. With antidepressant use, remission is achieved in about one-third of older patients, and relapse risk is reduced [21]. In general, efficacy is comparable across the various antidepressant types [22], and increases with severity of the depression [23]. Expert-based consensus guidelines recommend that in older persons, treatment duration for a first episode of depression is at least 1 year after achieving remission [8]. If there is no indication (anymore), the antidepressant should be withdrawn. Off-label antidepressant use is highly prevalent, especially among those with dementia living in long-term care institutions [24]. In these elderly persons, antidepressants are frequently prescribed for behavioral and psychological symptoms of dementia (BPSD), such as anxiety, agitation, and sleeping disorders. Treating depression with antidepressants in demented patients, has no proven benefit compared to non-pharmacological treatment modalities (e.g. daytime activities) [8]. Likewise, for other (general) populations, there is insufficient evidence that antidepressants are superior to placebo for treating insomnia [25]*.* In fact, cognitive and behavioral therapy for insomnia (CBT-I) is generally effective in primary, and secondary insomnia (in patients with comorbid depression): it improves depression and anxiety symptoms [26]. Therefore, a trial of CBT-I should be considered as a first-line treatment option in insomnia in older people persons with increased fall risk.

### Antidepressant dosage

For most antidepressants, lowest effective and maximal dose for geriatric patients have been established (SmPCs and handbooks). Dose adjustment can be indicated in case of decreased glomerular filtration rate in patients using drugs that have a small therapeutic index and are cleared renally (e.g. lithium) [27]. SSRI associated fall risk appears to be dose-dependent, whereas for other antidepressants dose-dependency is less evident from the available literature [5]. If response to adequately dosed first-line antidepressant therapy is too limited, switching to another antidepressant (class) can be considered [8]. Practical cross-titration recommendations for switching from one antidepressant to another, are available online. For example, the tables provided on www.switchwiki.eu are used by professionals from over 130 countries worldwide.

In current clinical practice, despite existing guidelines advising a stepwise order, the next line(s) of antidepressant therapy are frequently selected by means of educated trial-and-error, and/or titrated through therapeutic drug management (TDM). This approach is associated with disappointing success rates [28], delays clinical improvement and increases the risks for patients. Evidence is growing that genetic variation in cytochrome P450 enzymes contributes to variation among patients in the adverse effect toxicity profile of antidepressants and their effectiveness for major depression [29]. Guidelines for customized dosing of TCAs and SSRIs based on patients’ CYP2C19 and CYP2D6 polymorphisms have been designed by the Clinical Pharmacogenetics Implementation Consortium (CPIC). With regard to the place of genetic testing in guiding antidepressant choice, there is currently no global consensus [30]. In clinical practice, pharmacogenetic testing is often applied to patients who are intolerant to antidepressants or have failed to respond. Recent studies that applied pharmacogenomics to guide antidepressant choice show promising results: combinatorial testing of a total of 59 pharmacokinetic and pharmacodynamics alleles and gene variants in older adults suffering from major depressive disorder, significantly improved response and remission rates at week 8 compared to treatment as usual (difference of 14 and 13%, respectively) [31]. Thus, it is likely that in the near future, there will be a shift towards a more personalized approach in antidepressant therapy by applying pharmacogenomics.

If switching to another agent also fails to achieve remission, add-on therapy should be considered. Lithium augmentation is effective in older adults, with an overall response rate of 42% (95% CI; 21–65%) [8]. Of note, however, lithium treatment in older patients should be closely monitored due to the high risk for lithium toxicity due to its narrow therapeutic index, particularly if accompanied by low salt intake, diuretic use or dehydration. In case of severe (psychotic and/or life-threatening) or refractory depression, electroconvulsive therapy (ECT) has been shown highly effective in older persons, reaching efficacy rates of 60–80% [8]. Compared to pharmacological therapy, ECT has been associated with better efficacy and less side effects [32]. In selected patients, including those with comorbidities that constitute contra-indications for antidepressants, and in case of high suicidality or psychotic depression, ECT may even represent the treatment of choice. Also, addition of atypical antipsychotics in treatment resistant depression occurs in clinical practice. A recent RCT (*n* = 181, mean age 66 years old) showed that adding an atypical antipsychotic (aripiprazole) to an antidepressant (venlafaxine) resulted in remission in 44% of the participants compared to 29% percent in the (add-on) placebo group (NNT 6.6; 95%CI 3.5–81.8) [8]. Of note, however, the use of atypical antipsychotics in older adults is also associated with increased fall risk [4]. Therefore, adding these drugs to antidepressants in fall-prone older adults should be performed with great caution.

## Fall-related adverse effects of antidepressants

Our recent systematic review and meta-analysis [5], including 248 studies, showed that antidepressant use significantly increased fall risk (pooled OR 1.57 (95% Cl 1.43–1.74). Fall risk increased the most with the use of SSRIs and TCAs (OR 2.02; 95% CI 1.85–2.20 and OR 1.41; 95% CI 1.07–1.86, respectively. Fall risk appears to be most pronounced in the first weeks after initiating antidepressant use. Possibly, this is partly related to the fact that antidepressant-associated and depression-associated fall risks are simultaneously present in this timeframe [9]. Besides, it has been shown that side effects such as orthostatic hypotension and hyponatremia are strongest upon starting or increase of dosage. This may also affect antidepressant-related fall risk over time, since antidepressant-related side effects relevant to falling can be present within hours to days after treatment initiation [33], whereas optimal pharmacological response is most likely to be observed only after several weeks of therapy [34]. A vast amount of antidepressant side effects can contribute to (or cause) falls in older people, mainly being sedation, impaired balance/reaction time, orthostatic hypotension, cardiac conduction and rhythm disorders, and drug-induced movement disorders [9]. Most of the adverse effects are predictable from their relative receptor affinity. Cardiac adverse effects (e.g. arrhythmia and conduction delay), are caused by ion channel blockade [35] and, therefore, not predictable based on their receptor affinity.

Unfortunately, most of the evidence on antidepressant-related fall risk in older adults originates from observational studies comparing antidepressant users versus non-users; published data on antidepressant head-to-head comparison studies in older adults are scarce. Therefore, the evidence in this field may be biased through confounding by indication. It is, however, well established that adverse effect profile and tolerability differs substantially between different (groups of) antidepressants (Table 1). In clinical practice, selecting an antidepressant in older adults having increased fall risk should be individualized, guided by both antidepressant-specific adverse effect and tolerability profiles and patient characteristics. In the following paragraphs, an overview is provided of fall-related side effects of antidepressants, and antidepressant drug–drug interactions.

**Table 1:** Prevalence of fall-related side effects of antidepressants

	Orthostatic hypotension	Imbalance and/or dizziness	Extrapyramidal symptoms	Sedation	Delirium or confusional state	Visual impairment	Hyponatremia
*SSRIs*							
Citalopram Escitalopram Paroxetine Fluvoxamine Fluoxetine Sertraline	No data No data + + + + + + No data	+ + + + + + + + + + + + + + + + + + +	No data No data + + + + No data + + +	+ + + + + + + + + No data + + + + + + +	+ + + + + + + + + No data +	No data + + + + + No data + + + + + +	+ + + + + +
*SNRIs*							
Venlafaxine Duloxetine	+ + + +	+ + + + + + +	No data +	+ + + + + + + +	+ +	+ + + + + +	+ +
*TCAs*							
Amitriptyline Nortriptyline Clomipramine Doxepin Maprotiline Dosulepin	+ + + + + + + + + + + + + + + + + + +	+ + + + + + + + + + + + + + + + + + + + + +	No data No data No data + + + No data + +	+ + + + No data + + + + + + + + + + + +	+ + + + + + + + + + + +	+ + + + + + + + + + + + + + + + + + +	+ + + No data No data + + No data
*Other*							
Mirtazapine Bupropion Trazodone Agomelatine Vortioxetine Mianserin	+ + + + + + + No data No data + + +	+ + + + + + + + + + + + + + + + No data	No data + No data No data No data +	+ + + + No data + + + + + + No data + + + +	+ + + + + + + + + + No data No data	No data + + + + + + + + + No data	+ No data No data No data + +

+ : < 1/1000

+  + : 1/100–1/1000

+  +  + : 1/10–1/100

+  +  +  + : > 1/10

Source: antidepressant SmPCs

### Sedation, sleep disturbance, delirium

Sedation (sleepiness, drowsiness, or somnolence) is a frequent side effect of antidepressants and increases fall risk by impaired reaction capability [36]. Many antidepressants negatively influence sleep quality through rapid eye movement (REM) sleep suppression (TCAs, MAOIs, SSRIs, venlafaxine, bupropion, and trazodone) [9]. The sedative effect is caused by antihistaminergic, antinoradrenergic, serotonergic, and alpha-1 blocking effects of antidepressants [37]. TCAs, tetracyclics and trazodone are potent histamine receptor antagonists, causing sedation and daytime drowsiness. Due to its sedative effect, mirtazapine is frequently used in depressed patients with insomnia or in agitated depression. In older persons, however, mirtazapine should be prescribed with caution: a large observational study found that mirtazapine use was associated with a higher adverse event rate than other antidepressants [6]. One of the newest antidepressants, agomelatine, is an antidepressant with unique pharmacological features; it is a melatonin agonist and a selective serotonin antagonist [38]. The first antidepressant with activity on melatonin and serotonin receptors. Although studies suggest that agomelatine improves sleep (through restoration of the circadian rhythm) and has antidepressant effects, literature on patients over 75 years of age is scarce. However, there are concerns over the potential for agomelatine-induced hepatotoxicity. Also, agomelatine use is associated with sleepiness. Therefore, agomelatine is not to be considered a drug of first choice in older (fall-prone) patients.

SSRIs (and related drugs) have alerting properties and may cause nocturia, impairing sleep and subsequently daytime drowsiness [9]. Theoretically, nocturia might also increase fall risk in long-term lithium use complicated by nephrogenic diabetes insipidus [39]. However, literature on lithium-associated fall risk is too scarce to draw firm conclusions on this topic [17]. Furthermore, all antidepressants potentially contribute to delirium, a risk factor for falling in itself.

### Orthostatic hypotension and dizziness

Orthostatic hypotension (OH; a drop of at least 20 mmHg in systolic or 10 mmHg diastolic blood pressure upon standing within the first 3 to 5 min [40]) is highly prevalent in community-dwelling older persons, nursing home residents and hospitalized older people (prevalence of 10–30, 50 and 67%, respectively) [41]. OH is a well-established fall-risk factor that doubles the risk of falling in nursing home residents with a history of previous falls [42]. It results in transient cerebral hypoperfusion upon standing, which may result in a fall and/or syncope. Often, patients with OH report dizziness or lightheadedness upon standing. Other symptoms of hypoperfusion include blurred vision or in severe cases coat hanger pain [43]. Depending on the speed of the drop in blood pressure and its recovery, however, it can also be asymptomatic. Frequently, older persons do not recall a syncopal event: over 50% of those with an observed transient loss of consciousness did not remember the loss of consciousness. OH is also a frequently prevalent adverse drug reaction in antidepressants, reported predominantly for TCAs, early generation monoamine oxidase inhibitors and SNRIs, and to a lesser extent for SSRIs [44]. TCAs and tetracyclics (e.g. mirtazapine and mianserin) are notorious for causing (orthostatic) hypotension [35]. For this reason, slow dose titration is indicated for these medications. However, SSRIs can also cause OH through peripheral vascular effects and/or the ability to induce bradycardia [35]. Gastrointestinal symptoms (e.g. loss of appetite, nausea) are other frequent side effects of SSRIs, potentially leading to decreased intake of fluids and/or food. In turn, dehydration and deconditioning may occur, contributing to both OH and mobility related fall risk. Dizziness (also without OH) is a frequent reported side effect of all antidepressants and an important fall risk factor (Table 1).

### Anticholinergic effects

Anticholinergic medication exposure increases fall risk directly through negative effects on cognitive and physical functioning, because it may cause postural hypotension, impaired cognitive performance, arrhythmia, QT prolongation, and blurred vision [45]. Anticholinergic drugs may also indirectly increase fall risk because they may worsen common conditions associated with increased fall risk, such as dementia, diabetes, and Parkinson’s disease. The anticholinergic potential of TCAs is well established. In older persons, nortriptyline is preferred over amitriptyline due to its less anticholinergic potential [46].

### Movement disorders

TCAs, SSRIs, and lithium have dopamine blocking effects, and can, therefore, induce drug-induced Parkinsonism, characterized by tremor, bradykinesia, hypokinesia, rigidity, and postural instability. Drug-induced Parkinsonism can occur both shortly after antidepressant initiation (within days), and in chronic use (after months), albeit to a lesser extent. The prevalence of these movement disorders associated with SSRI use in older persons is approximately 10% [47]. Extrapyramidal symptoms have also been reported with lithium therapy [48]. Furthermore, long-term lithium use is also associated with cerebellar ataxia [39], which theoretically also increases fall risk.

### Cardiac rhythm and conduction disorders and other cardiovascular side effects

Antidepressant use (mainly TCAs, tetracyclics and SNRIs, and to a lesser extent SSRIs [44]) is frequently associated with cardiovascular side effects such as arrhythmia and conduction delays [49]. The cardiovascular effects and toxicity of TCAs have been well documented in depressed patients, including those without pre-existing cardiac disease [35]. Most frequently, TCAs prolong PR, QRS, and QT intervals. Although these side effects are observed at therapeutic doses, related morbidity and mortality is predominantly observed in case of toxic levels. Also, TCAs have been shown to increase risk of sudden cardiac death [35]. Therefore, TCAs must be used with great caution in patients with cardiac disease.

Although newer generation antidepressants were initially reported to have fewer and less severe cardiovascular adverse effects, current evidence shows that clinically relevant cardiovascular (event) risk is equal for TCAs, SSRIs and other antidepressants [35, 50]. SSRI use (especially (es-) citalopram) is associated with QT interval prolongation and heart rate abnormalities (both brady- and tachycardia) [37]. Therefore, combined use of (es-)citalopram and other QT interval increasing drugs should be avoided. Fluoxetine therapy is frequently complicated by development of first-degree atrioventricular and atrioventricular block [35]. Although venlafaxine has been associated with QT interval prolongation, tachycardia and palpitations [51], the SNRIs are generally relatively safe with respect to rhythm and conduction disorders [12]. Nevertheless, there have been concerns regarding its effect on increasing blood pressure related to noradrenergic effects. In older persons, however, the overall risk for venlafaxine-induced hypertension seems low [49].

Bupropion and mirtazapine also appear to have little cardiovascular side effects [49]. Also, the newest generation antidepressants (e.g. agomelatine and vortioxetine) appear to be safe regarding cardiovascular effects in adults, but literature on older and vulnerable individuals is limited. As mentioned above, SSRIs and TCAs are also associated with autonomic dysfunction, which has been linked to an increased risk of developing new-onset atrial fibrillation in a large cohort study [52]. Also, increased serotonin receptor activation has been associated with increased cardiovascular risk (mainly atrial fibrillation) [50]. Chronic lithium use (monotherapy) has been linked to atrial and ventricular instability, not only in case of toxic blood concentrations, but also in case of therapeutic blood concentrations [39].

### Hyponatremia

In older people, hyponatremia is a frequently occurring and potentially serious adverse drug effect of antidepressants [53]. Hyponatremia increases fall risk by causing confusion, sedation, dizziness or muscle weakness, even if the hyponatremia is of mild severity (serum concentration < 135 mEq/L) [54]. It can be caused by the syndrome of inappropriate antidiuretic hormone secretion (SIADH), and results from administering drugs/antidepressants with serotonergic action, such as SSRIs or TCAs [55]. SSRI-induced hyponatremia occurs in 10–15% of older adults. Paroxetine has the highest incidence of SSRI associated hyponatremia [56]. In psychiatric inpatients, ~ 10% of the patients using antidepressants were diagnosed with SIADH, particularly when using an SSRI or venlafaxine [57]. The risk of developing hyponatremia is highest after initiating antidepressants or increasing the dose. Therefore, international guidelines recommend to check serum sodium levels prior to and within the first weeks of treatment initiation, and in case of acute conditions commonly associated with hyponatremia (e.g. inadequate salt and/or protein intake). Comorbidities such as chronic renal failure and hypertension, and concurrent use of other drugs with a propensity to cause hyponatremia (e.g. diuretics) increase the risk of developing hyponatremia while using antidepressants. In individuals at increased risk for hyponatremia based on these comorbid conditions, newer generation antidepressants (with the exception of duloxetine, mirtazapine, and trazodone) should be avoided or closely monitored [12].

### Bone quality and fracture risk

Literature shows that SSRI and TCA use may negatively impact bone mineral density, and increase (hip) fracture risk [58]. This increased fracture risk potentially negatively affects the impact of falls, by increasing the risk of fall-related injury. For SSRIs, preclinical data suggest that serotonergic pathways negatively affect metabolism, but the pathophysiological mechanisms have not been completely elucidated [59], and clinical evidence for decreased bone mineral density due to SSRIs, has been inconsistent [60]. TCAs may also increase fracture risk, but through mechanisms other than a direct effect on bone mineral density [61]. Based on current (but scarce) evidence, antidepressants other than SSRIs and TCAs do not appear to increase fracture risk [61].

#### Drug–drug interactions

In general, antidepressants are characterized by having a high risk of drug–drug interactions, obviously limiting treatment options in older individuals with comorbidity and polypharmacy. The tendency to cause drug–drug interactions however, varies considerably between antidepressants. In general, older compounds, such as TCAs and MAOIs, have a higher potential for these interactions than newer compounds [62]. Of the newer antidepressants, fluoxetine, fluvoxamine, and paroxetine have high risk of drug–drug interactions, whereas citalopram and sertraline have a low inhibitory activity on different drug metabolizing enzymes [62]. Of clinical relevance is the fact that initiation of antidepressants that potently inhibit the CYP2D6 enzyme (e.g. fluoxetine, paroxetine) in patients already on certain beta-blockers (CYP2D6 substrates), can lead to accumulation of the beta-blocker and associated bradycardia, hypotension, syncope, and falls [63]. With antidepressants exhibiting weak CYP2D6 inhibition (e.g. sertraline, citalopram, fluvoxamine, or venlafaxine), this risk is significantly lower. Therefore, these antidepressants are preferred in older patients already on these beta-blockers. In case of SSRI overdose, or combined use of highly sedative antidepressants and St John’s Wort (*Hypericum Perforatum*) or lithium, there is a risk of developing the potentially life-threatening serotonin syndrome, albeit rare [39]. This syndrome theoretically increases fall risk through causing delirium, hypertonia, and autonomic dysregulation.

## Deprescribing antidepressants

In general, older adults with polypharmacy are open to medication deprescribing if their doctor thinks it appropriate [64]. Achieving permanent antidepressant withdrawal, however, is notoriously challenging [65]. A key element to successful deprescribing is to work in close collaboration with the patient and/or caregivers, taking into account patient’s goals, values, attitudes and preferences (shared decision-making) [16]. Their beliefs and fears should be explored and addressed. Most antidepressant users fear withdrawal symptoms, insomnia and relapse of depression, especially if they have taken these medications for many years [64]. Withdrawal/discontinuation symptoms (e.g. severe anxiety, mood lability, low mood, and dizziness) have been described for antidepressants with serotonergic effects, and cholinergic rebound symptoms (e.g. anxiety, insomnia) have been reported for anticholinergic antidepressants (mainly TCAs) [66]. Because these symptoms may overlap with depressive symptoms, they may be misinterpreted as recurrence of depression. As a result, (previous) antidepressant cessation attempts may be erroneously labeled as failed. Patients should be reassured and informed that careful withdrawal of antidepressants with patient education and close monitoring appears safe and usually does not cause harm [16, 65]. Indeed, slow tapering (over weeks to months) of antidepressants minimizes the prevalence and severity of withdrawal symptoms, especially with agents with short half-lives (e.g. paroxetine and venlafaxine) [8]. Agomelatine appears to be devoid of clinically relevant withdrawal symptoms [67], and therefore, the drug labeling indicates that it can be stopped abruptly.

Literature on how to taper antidepressants is scarce and international guidelines generally do not provide detailed guidance with regard to dosing steps and rate of tapering. Instead, they recommend “slow” tapering. In current clinical practice, dose tapering in weekly steps is often performed for SSRI/SNRIs, based on their elimination half-life. All SSRIs and SNRIs (except from fluoxetine and vortioxetine) have elimination half-lives not exceeding 40 h, and, therefore, steady-state concentration is reached within 1 week after dose adjustment. Slow tapering, however, may be challenging because antidepressants come in only a limited number of registered dosages that do not allow for flexible and individualized slow and gradual tapering over time [68]. Especially for antidepressants for which no liquid formulations exist (e.g. venlafaxine and sertraline) this may be problematic. For some antidepressants, however, customized tapering strips have been developed, allowing for gradual dosage reduction. In an observational study, these strips were effective in achieving a gradual dosage reduction in 71% of participants (individuals who had experienced a failed antidepressant cessation attempt because of severe withdrawal symptoms previously) [68].

Another aspect to consider is the timing of deprescribing. Stress-inducing circumstances and insomnia reduce likelihood of successful antidepressant withdrawal [69]. Thus, insight in (potential) psychosocial stressors and levels of social support are required before deprescribing. These elements should be considered in scheduling of antidepressant withdrawal, and should be part of the individualized deprescribing process. Also, patients may be reluctant to withdraw antidepressants because they have little faith in non-pharmacological interventions. They should be informed that literature proves otherwise: non-pharmacological interventions (e.g. cognitive behavioral therapy, mindfulness-based cognitive therapy, and physical exercise) have proven efficacy both in treatment for depression on top of antidepressants [70], and in helping patients discontinue antidepressants without increasing the risk of relapse/recurrence [71]. Indeed, patient education empowers them to actively engage in deprescribing decisions and enhances success rates [72].

Also among prescribers there is reluctance to deprescribe antidepressants, despite the fact that its use is a well-established and common fall-risk factor, and deprescribing of FRIDs and specifically antidepressants is an effective means of reducing fall risk [73]. This reluctance appears to be based on lack of confidence regarding if and how to withdraw antidepressants [74]. Tools and resources, are available to help prescribers in the deprescribing process [16]. These will be discussed in the next paragraph.

## Decision aids in deprescribing

**Fig. 1:**
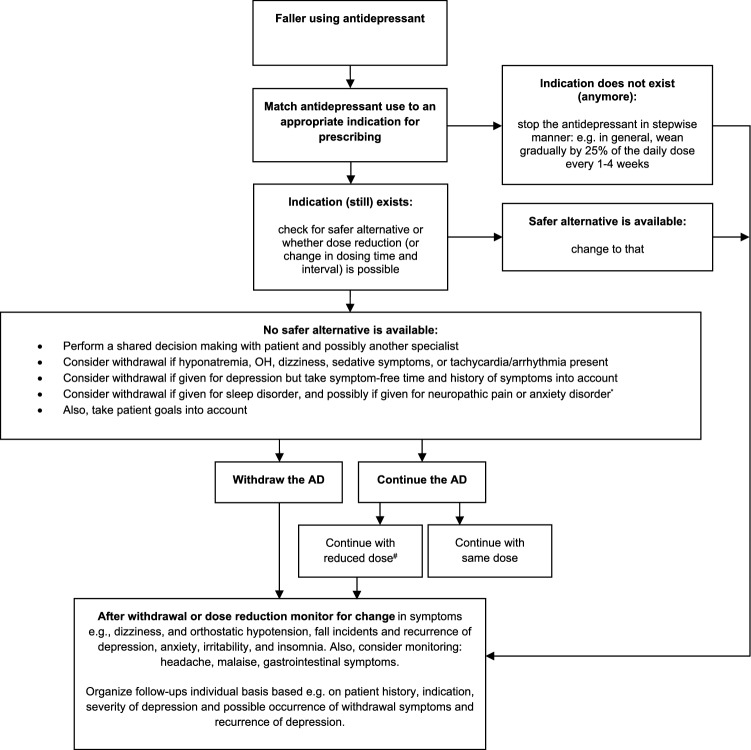
Decision tree for antidepressant withdrawal among fallers [77]. ^*^ Of note: if the antidepressant was prescribed for an anxiety disorder, the relapse risk of the anxiety disorder is high in adults [78]. ^#^ For TCAs, it is recommended to pursue therapeutic blood concentrations and avoid subtherapeutic dosing [79]

A number of tools are available to assist clinicians in deprescribing potentially inappropriate medications in older people, including FRIDs [75]. These tools include among others the STOPP (Screening Tool of Older Persons’ Potentially Inappropriate Prescriptions) criteria [76]. Recently, the European Geriatric Medicine Society (EuGMS) Task and Finish Group on FRIDs have, through an European expert Delphi consensus effort, developed the STOPPFalls tool [77]. This is a deprescribing tool that specifically focuses on falls prevention and includes practical deprescribing algorithms for different drug classes. As an example, the antidepressant withdrawal algorithm of STOPPFalls is depicted in Fig. 1 (also available on the website of the EuGMS Task and Finish group on FRIDs: https://www.eugms.org/research-cooperation/special-interest-groups/falls-and-fractures.html). The STOPPFalls tool is officially endorsed by the EuGMS and by the STOPP-START group. Whether implementation of this tool in clinical practice translates to reduction in fall risk is an area of ongoing research.

## Conclusions

Depression and antidepressant uses are common in older people. Both untreated depression and antidepressant use contribute to fall risk. Depression increases fall risk through psychomotor retardation, deconditioning, gait/balance abnormalities, impaired sleep/attention and fear of falling. Antidepressants are FRIDs and contribute to (or cause) falling through causing sedation, impaired balance/reaction time, OH, hyponatremia, cardiac conduction delay/arrhythmia, and/or drug-induced Parkinsonism.

Falling is an important driver of morbidity and mortality that requires prevention in older people. To minimize fall risk, it is important to make a personalized assessment, prescribe antidepressants with caution, and deprescribe antidepressants when the (potential) risks of antidepressant use outweighs the (potential) benefits.

A major barrier for deprescribing antidepressants is the high level of complexity of deprescribing antidepressants in older persons with multiple comorbidities and medications. However, detailed insight in fall-related side effect profile of the different antidepressants and the newly developed expert-based decision aid assist in clinical decision-making.

## Abbreviations

BPSD: Behavioral and psychological symptoms of dementia
CBT-I: Cognitive and behavioral therapy for insomnia
CI: Confidence interval
CPIC: Clinical Pharmacogenetics Implementation Consortium
CYP: Cytochrome P450
DDI: Drug–drug interaction
ECT: Electroconvulsive therapy
EuGMS: European Geriatric Medicine Society
FRID: Fall-risk increasing drug
MAOI: Monoamine oxidase inhibitor
OH: Orthostatic hypotension
OR: Odds ratio
QoL: Quality of life
RaR: Relative attributable risk
REM: Rapid eye movement
SIADH: Syndrome of inappropriate antidiuretic hormone secretion
SmPC: Summary of product characteristics
SNRI: Serotonin noradrenalin reuptake inhibitor
SSRI: Selective serotonin reuptake inhibitor
START: Screening Tool to Alert doctors to Right Treatment
STOPP: Screening Tool of Older Persons’ Potentially Inappropriate Prescriptions
TDM: Therapeutic drug management
TCA: Tricyclic antidepressant
